# Test-retest reproducibility of cardiac magnetic resonance imaging in healthy mice at 7-Tesla: effect of anesthetic procedures

**DOI:** 10.1038/s41598-017-07083-1

**Published:** 2017-07-27

**Authors:** Michael Joubert, Pia Tager, Damien Legallois, Estelle Defourneaux, Bastien Le Guellec, Bernhard Gerber, Remy Morello, Alain Manrique

**Affiliations:** 1 EA4650, Université de Caen Normandie, 14000 Caen, France; 2 Nuclear Medicine, CHU de Caen, 14000 Caen, France; 3 Cardiology, CHU de Caen, 14000 Caen, France; 4 Cardiology, Université Catholique de Louvain, B-1348 Louvain-la-Neuve Brussels, Belgium; 5 Biostatistic, CHU de Caen, 14000 Caen, France; 6 Diabetes Care Unit, CHU de Caen, 14000 Caen, France

## Abstract

Cardiac magnetic resonance (CMR) has emerged as a powerful tool for *in vivo* assessments of cardiac parameters in experimental animal models of cardiovascular diseases, but its reproducibility in this setting remains poorly explored. To address this issue, we investigated the test-retest reproducibility of preclinical cardiac magnetic resonance imaging (CMR) at 7 Tesla in healthy C57BL/6 mice, including an analysis of the impact of different anesthetic procedures (isoflurane or pentobarbital). We also analyzed the intra-study reproducibility and the intra- and inter-observer post-processing reproducibility of CMR images. Test-retest reproducibility was high for left ventricular parameters, especially with the isoflurane anesthetic procedure, whereas right ventricular parameters and deformation measurements were less reproducible, mainly due to physiological variability. Post-processing reproducibility of CMR images was high both within and between observers. These results highlight that anesthetic procedures might influence CMR test-retest reproducibility, an important ethical consideration for longitudinal studies in rodent models of cardiomyopathy to limit the number of animals used.

## Introduction

Heart failure resulting from ischemic or non-ischemic cardiomyopathy is one of the most important causes of death worldwide. In the past two decades, many animal models have been developed to explore heart failure and cardiovascular diseases. New cardiac imaging technologies that permit non-invasive assessment of cardiac function have opened the field of longitudinal analysis of functional changes after therapeutic interventions in these various animal models. Cardiac magnetic resonance (CMR) offers a high degree of intrinsic tissue contrast from tissue relaxation times and bulk flow that is used to obtain volumetric data on heart chambers, myocardial mass, global and regional function, myocardial strain, perfusion and tissue characteristics^1, 2^. In humans, due to its high reproducibility, CMR is considered a gold standard for non-invasive assessment of both left and right ventricular function^3, 4^ and left ventricular strain^5^. However, CMR in small animals remains challenging and not well standardized^1^. The small masses of the mouse body and heart (respectively 20–40 g and 50–135 mg in normal mice) and the high cardiac and respiratory rates of anesthetized animals raise several technical issues. Using dedicated high-field small animal systems, CMR allows dual cardiac and respiratory gated imaging of a fast-beating mouse heart^1^. The recent availability of self-gated cardiac imaging^6^ has dramatically simplified data acquisition of high temporal resolution images covering the entire ventricular volume. However, even though recent investigations have suggested excellent intra- and inter-observer reproducibility of cardiac function and left ventricular mass measurement using self-gated CMR, longitudinal reproducibility remains poorly investigated^7^. While the feasibility of CMR tagging has been demonstrated in mice for evaluating regional myocardial wall strain^8, 9^, this approach is based on spatial modulation of magnetization applied to a single-slice ECG-gated fast 2D gradient echo sequence and may be compromised by limited temporal resolution.

Finally, anesthetic drugs and the depth of anesthesia may lead to complex cardiovascular effects through the modulation of heart and respiratory rates or through direct cardiovascular effects, which may potentially impact cardiac functional assessment.

The aim of this study was to assess the reproducibility of CMR in a preclinical setting. We evaluated the inter-/intra-study and inter-/intra-observer reproducibility of CMR in healthy mice using a standardized imaging protocol with two different anesthetic procedures.

## Results

### Physiological measurements

**Figure 1:**
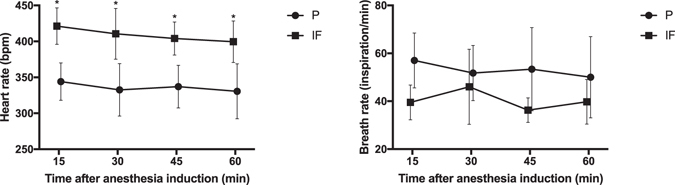
Heart rate (left panel) and breath rate (right panel) profiles during CMR examinations. Data are the mean ± SD (error bars) of HR and BR in animals from groups IF (isoflurane) (black squares) and P (pentobarbital) (black circles), measured every 15 minutes after anesthesia induction throughout the CMR procedure. *p < 0.05 between groups.

Twenty mice were employed (ten mice in each anesthetic group). Physiological parameters are summarized in Table 1. Mean body weight was not different between the isoflurane (group IF) and pentobarbital (group P) groups during the first CMR exam (CMR1) (27.9 ± 0.84 and 28.4 ± 1.41 g, respectively). Mean body weight was stable in both groups IF and P at the second CMR exam (CMR2) one week later. In group IF, mean heart rate (HR) and breath rate (BR) were also not significantly different between CMR1 and CMR2. Conversely, in group P, mean HR was stable between examinations, but mean BR was higher during CMR2 than during CMR1 (58 ± 17 compared to 48 ± 8 inspiration/min, respectively; p = 0.0155). HR was significantly higher and BR significantly lower in animals in group IF compared with group P during both CMR examinations (Table 1). HR and BR profiles throughout the CMR examinations are presented in Fig. 1. HR was higher in animals from group IF at all time points (Fig. 1). Intra-study HR and BR variability (expressed as intra-study standard deviation) were not different between groups (Table 1).

**Table 1:** Physiological parameters during cardiac magnetic resonance.

	Group IF			Group P			
	CMR1	CMR2	Inter-study comparison (p)	CMR1	CMR2	Inter-study comparison (p)	Inter anesthetic procedure comparison (p)
Mean HR (bpm)	408 (25)	410 (32)	0.7685	331 (30)	341 (33)	0.2580	<0.0001
Intra-study HR SD (bpm)	18 (7)	24 (10)	0.2257	17 (11)	17 (8)	0.9834	0.1863
Mean BR (inspiration/min)	39 (8)	41 (13)	0.6505	48 (8)	58 (17)	0.0155	0.0470
Intra-study BR SD (inspiration/min)	6 (3)	6 (7)	0.9833	5 (2)	6 (3)	0.7279	0.7097
Body weight (g)	27.9 (0.84)	27.5 (1.31)	0.3536	28.4 (1.41)	28.3 (1.67)	0.7627	0.1848

Mean heart rate (HR), intra-study HR standard deviation (SD), mean breath rate (BR), intra-study BR SD and body weight during first (CMR1) and second (CMR2) cardiac magnetic resonance exams are presented for both anesthetic procedures (IF – isoflurane and P – pentobarbital). Data are the mean (SD).

### Test-retest reproducibility

**Figure 2:**
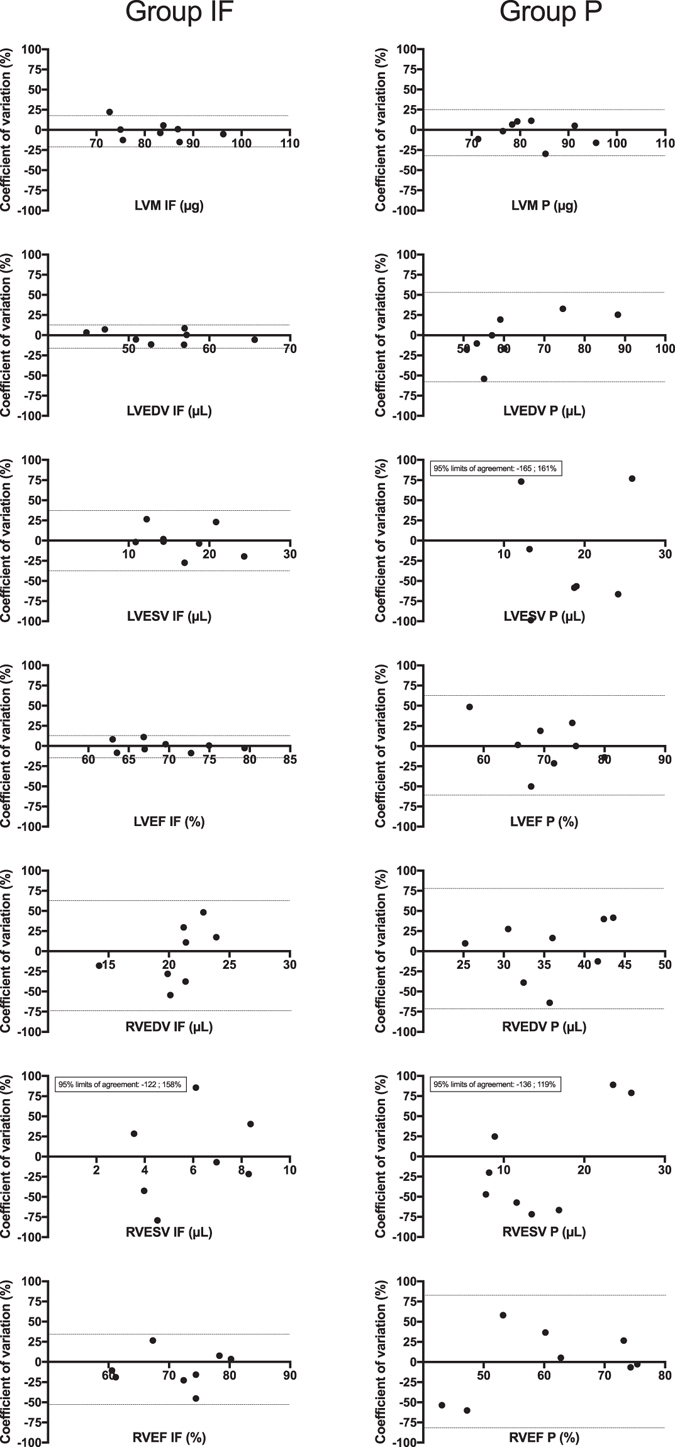
Bland-Altman representation of inter-study differences. Mean CMR1/CMR2 values on the X-axis are plotted against the coefficient of variation (COV) between CMR1 and CMR2 on the Y-axis for groups IF (isoflurane) (left column) and P (pentobarbital) (right column). The 95% limits of agreement are represented on each graph by thin dotted lines (or printed values when out of the graph range).

In group IF, test-retest reproducibility was high, with a low coefficient of variation (COV) between CMR1 and CMR2 for left ventricular (LV) function parameters that ranged from 5.36 ± 3.62 to 12.97 ± 9.64%. However, the measurements of right ventricular (RV) and strain parameters were fairly reproducible, except for right ventricular ejection fraction (RVEF) (test-retest COV of 15.96 ± 9.74%). In group P compared with group IF, test-retest COV was significantly higher for left ventricular end diastolic volume (LVEDV), left ventricular end systolic volume (LVESV), left ventricular ejection fraction (LVEF) and right ventricular end systolic volume (RVESV). In this group, the reproducibility threshold was only reached for left ventricular mass (LVM), LVEDV and LVEF (Table 2). Bland-Altman graphs also showed wider limits of agreement for group P (Fig. 2). In addition, intra-class correlations (ICCs) were only significant for LVEDV, LVESV and LVEF in group IF, whereas no ICC was significant in group P (Table 2). The results of pooled mean data from CMR1 and CMR2 were compared between the IF and P groups for all CMR parameters (Table 2).

**Table 2:** Inter-study reproducibility as evaluated by the absolute difference, coefficient of variation (COV) (expressed in %) and interclass correlation (ICC) between results from the first (CMR1) and second (CMR2) cardiac magnetic resonance exams, during isoflurane (IF) and pentobarbital (P) anesthesia.

	Group IF					Group P					
	CMR1	CMR2	Absolute difference	COV	ICC [95%CI] (p)	CMR1	CMR2	Absolute difference	COV	ICC [95%CI] (p)	p^†^
LVM (µg)	83.55 (7.22)	87.26 (13.67)	8.10 (8.58)	8.64 (8.36)	0.449 [−0.179–0.825] (0.074)	81.10 (8.75)	83.95 (11.35)	9.68 (7.58)	10.62 (7.42)	0.279 [−0.437–0.794] (0.219)	0.689
LVEDV (µL)^§^	53.92 (5.58)	54.20 (7.14)	3.96 (2.49)	7.00 (4.28)	0.737 [0.273–0.927] (0.003)	62.54 (20.53)	62.03 (8.28)	14.07 (10.30)	19.04 (12.62)	0.380 [−0.341–0.832] (0.142)	0.008
LVESV (µL)	16.15 (4.03)	15.84 (5.11)	2.40 (1.97)	12.97 (9.64)	0.779 [0.361–0.939] (0.002)	19.67 (12.58)	18.46 (8.30)	14.28 (9.35)	50.26 (19.73)	−0.349 [−0.807–0.406] (0.824)	0.001
LVEF (%)	70.23 (5.56)	71.12 (6.71)	3.82 (2.55)	5.36 (3.62)	0.729 [0.256–0.924] (0.004)	70.46 (10.26)	70.07 (14.05)	15.51 (11.99)	19.43 (15.06)	−0.324 [−0.797–0.429] (0.805)	0.008
RVEDV (µL)^§^	21.75 (5.95)	20.61 (3.73)	6.57 (3.04)	26.21 (10.32)	−0.089 [−0.638–0.536] (0.601)	36.64 (10.95)	35.25 (8.00)	11.56 (7.22)	26.13 (13.49)	−0.040 [−0.661–0.638] (0.535)	0.064
RVESV (µL)^§^	6.86 (2.81)	5.37 (2.30)	3.00 (2.64)	37.09 (24.43)	−0.123 [−0.658–0.511] (0.640)	15.20 (12.36)	13.86 (5.03)	9.55 (7.65)	42.98 (15.82)	0.157 [−0.535–0.741] (0.332)	0.022
RVEF (%)^§^	68.68 (10.05)	73.40 (11.81)	12.54 (8.84)	15.96 (9.74)	0.052 [−0.546–0.630] (0.433)	62.24 (19.22)	60.11 (13.13)	16.77 (11.63)	25.26 (18.61)	0.225 [−0.482–0.771] (0.267)	0.393
Ecc base^§^	−9.94 (2.80)	−10.33 (2.98)	2.85 (2.48)	45.13 (56.66)	0.143 [−0.545–0.734] (0.346)	−13.05 (3.75)	−12.64 (1.81)	2.67 (3.66)	23.64 (32.53)	0.539 [−0.151–0.885] (0.056)	0.912
Ecc apex^§^	−12.69 (2.85)	−11.86 (3.18)	3.80 (3.67)	55.12 (72.01)	−0.599 [−0.895–−0.103] (0.958)	−15.77 (2.35)	−14.70 (2.84)	2.52 (2.71)	18.25 (18.46)	0.462 [−0.250–0.860] (0.093)	0.505
Err base	5.05 (1.96)	7.10 (2.54)	3.01 (2.90)	32.62 (25.46)	−0.438 [−0.841–0.314] (0.833)	4.88 (4.00)	4.33 (2.14)	2.12 (1.85)	28.99 (19.22)	0.330 [−0.391–0.813] (0.179)	0.544
Err apex	7.38 (1.99)	4.91 (2.06)	3.29 (2.21)	40.35 (22.49)	0.449 [−0.845–0.301] (0.890)	6.47 (3.24)	7.58 (2.87)	4.56 (3.29)	50.42 (35.89)	0.447 [−0.268–0.855] (0.101)	0.387
Ell^§^	−10.65 (2.79)	−10.43 (2.24)	2.93 (3.55)	52.74 (90.08)	−0.758 [−0.948–−0.258] (0.994)	−14.08 (1.90)	−12.28 (2.13)	2.48 (2.10)	20.53 (15.04)	0.234 [−0.475–0.775] (0.259)	0.799

Data are the mean (SD) except for ICC [95% interval confidence] (p). ^†^Comparison of inter-study COV between groups IF and P. ^§^Indicates CMR parameters that are different (p < 0.05) between groups IF and P (pooled data from CMR1 and CMR2).

### Intra-study reproducibility of myocardial strain

For both groups IF and P, the intra-study COV of parameters from the two tagged sequences ranged from 15.43 ± 10.67 to 31.48 ± 25.37%, with no inter-group difference. The reproducibility threshold was obtained only for circumferential strain with both anesthetic procedures (Table 3). The ICCs were in line with this poor reproducibility as no ICC value reached significance (Table 3).

**Table 3:** Intra-study reproducibility as evaluated by the absolute difference, coefficient of variation (COV) (expressed in %) and interclass correlation (ICC) between results from the first (seq. 1) and second (seq. 2) tagged sequences within the same cardiac magnetic resonance exam during isoflurane (group IF) and pentobarbital (group P) anesthesia.

	Group IF					Group P					p*
	Seq. 1	Seq. 2	Absolute difference	COV	ICC [95%CI] (p)	Seq. 1	Seq. 2	Absolute difference	COV	ICC [95%CI] (p)	
Ecc base	−10.14 (2.82)	−10.71 (3.78)	1.81 (1.74)	19.60 (17.54)	0.143 [−0.545–0.734] (0.346)	−12.84 (2.79)	−13.22 (2.47)	1.86 (1.18)	15.43 (10.67)	0.539 [−0.151–0.885] (0.056)	0.931
Ecc apex	−12.27 (2.97)	−12.76 (2.59)	1.65 (1.43)	17.49 (19.06)	−0.599 [−0.895–0.103] (0.958)	−15.24 (2.52)	−14.31 (2.44)	2.78 (1.77)	22.23 (18.80)	0.462 [−0.250–0.860] (0.093)	0.070
Err base	6.08 (2.44)	6.47 (2.78)	2.45 (2.06)	29.51 (21.02)	−0.438 [−0.841–0.314] (0.883)	4.60 (3.04)	6.74 (4.68)	2.44 (2.99)	31.48 (25.37)	0.330 [−0.391–0.813] (0.179)	0.993
Err apex	6.15 (2.35)	7.33 (1.62)	1.78 (1.35)	23.95 (18.56)	−0.449 [−0.845–0.301] (0.890)	7.03 (2.95)	8.90 (5.15)	2.45 (3.02)	22.45 (18.14)	0.447 [−0.268–0.855] (0.101)	0.404
Ell	−10.54 (2.47)	−11.02 (2.76)	1.79 (2.03)	26.68 (46.65)	−0.785 [−0.948–−0.258] (0.994)	−13.18 (2.13)	−13.38 (4.04)	2.94 (3.08)	26.65 (29.06)	0.234 [−0.475–0.775] (0.259)	0.230

Data are the mean (SD) except for ICC [95% interval confidence] (p). *Comparison of intra-study difference between groups IF and P.

### Inter-observer reproducibility

Inter-observer post-processing COV was below 20% for all ventricular and strain analysis parameters except RVESV (29.77 ± 19.49%) and radial strain (Err base: 21.52 ± 13.03%) (Table 4). Accordingly, all ICC values approached +1 (perfect agreement) and were significant (Table 4).

**Table 4:** Inter-observer reproducibility as evaluated by the absolute difference, coefficient of variation (COV) (expressed in %) and interclass correlation (ICC) between results from the first (observer1) and second (observer2) observers who post analyzed the same CMR examinations.

	Observer1	Observer2	Absolute difference	COV	ICC [95%CI] (p value)
LVM (µg)	84.12 (10.37)	81.28 (10.00)	7.79 (5.29)	8.91 (5.81)	0.583 [0.321–0.762] (<0.001)
LVEDV (µL)	57.72 (11.68)	56.96 (10.44)	4.20 (4.63)	6.78 (6.90)	0.843 [0.715–0.917] (<0.001)
LVESV (µL)	17.36 (7.67)	17.67 (7.29)	2.38 (1.77)	13.22 (10.35)	0.922 [0.854–0.959] (<0.001)
LVEF (%)	70.49 (8.96)	69.61 (7.67)	3.65 (2.48)	5.06 (3.21)	0.861 [0.746–0.926] (<0.001)
RVEDV (µL)	27.74 (10.26)	23.64 (6.95)	5.69 (5.30)	18.09 (13.87)	0.630 [0.387–0.792] (<0.001)
RVESV (µL)	9.86 (7.58)	7.10 (5.47)	3.22 (3.40)	29.77 (19.49)	0.763 [0.584–0.871] (<0.001)
RVEF (%)	66.65 (14.09)	71.93 (12.57)	9.65 (6.85)	13.80 (11.45)	0.624 [0.378–0.788] (<0.001)
Ecc base	−15.49 (1.84)	−14.88 (1.22)	0.85 (0.82)	5.69 (5.33)	0.737 [0.188–0.940] (0.008)
Ecc apex	−15.87 (4.83)	−15.27 (4.81)	0.70 (0.52)	5.01 (3.61)	0.984 [0.932–0.997] (<0.001)
Err base	5.01 (2.41)	6.02 (2.09)	1.19 (0.69)	21.52 (13.03)	0.826 [0.398–0.962] (0.002)
Err apex	5.47 (1.95)	5.70 (1.87)	0.65 (0.68)	10.91 (9.72)	0.885 [0.566–0.975] (<0.001)
Ell	−13.64 (2.35)	−14.28 (2.77)	0.79 (0.56)	5.60 (3.56)	0.933 [0.729–0.986] (<0.001)

Data are the mean (SD) except for ICC [95% interval confidence] (p value).

### Intra-observer reproducibility

Reproducibility of intra-observer post-processing was good, with COV below 20% for all measured parameters and positive and significant ICC values for all parameters except LVM and right ventricular end diastolic volume (RVEDV) (Table 5).

**Table 5:** Intra-observer reproducibility as evaluated by the absolute difference, coefficient of variation (COV) (expressed in %) and interclass correlation (ICC) between results from the first (analysis1) and second (analysis2) post-processing of data from the same CMR examinations by the same observer.

	Analysis1	Analysis2	Absolute difference	COV	ICC [95%CI] (p value)
LVM (µg)	80.50 (8.00)	77.61 (7.35)	6.37 (6.97)	7.50 (7.55)	0.285 [−0.355–0.754] (0.187)
LVEDV (µL)	54.65 (7.06)	51.37 (7.24)	4.01 (4.53)	6.90 (6.79)	0.675 [0.153–0.907] (0.009)
LVESV (µL)	17.44 (5.36)	17.09 (5.39)	2.95 (3.81)	12.91 (13.28)	0.616 [0.054–0.887] (0.018)
LVEF (%)	68.47 (6.76)	67.25 (5.88)	4.45 (3.35)	6.37 (4.92)	0.623 [0.066–0.889] (0.016)
RVEDV (µL)	21.63 (4.73)	19.81 (3.79)	2.64 (3.61)	10.70 (13.46)	0.503 [−0.111–0.846] (0.050)
RVESV (µL)	6.05 (2.92)	6.64 (3.16)	1.30 (1.19)	17.76 (17.78)	0.839 [0.502–0.957] (<0.001)
RVEF (%)	72.18 (12.03)	67.17 (11.83)	5.76 (5.51)	7.99 (7.03)	0.795 [0.398–0.944] (0.001)
Ecc base	−12.84 (2.79)	−12.85 (2.64)	0.34 (0.39)	3.01 (3.57)	0.983 [0.938–0.996] (<0.001)
Ecc apex	−15.24 (2.52)	−15.02 (2.27)	1.01 (0.57)	6.89 (3.90)	0.886 [0.627–0.970] (<0.001)
Err base	4.60 (3.04)	4.68 (2.63)	0.91 (1.12)	16.70 (17.85)	0.879 [0.608–0.968] (<0.001)
Err apex	7.03 (2.95)	7.22 (2.81)	0.95 (0.87)	13.93 (11.65)	0.904 [0.681–0.975] (<0.001)
Ell	−13.18 (2.13)	−13.36 (2.33)	0.44 (0.34)	3.49 (2.51)	0.970 [0.891–0.992] (<0.001)

Data are the mean (SD) except for ICC [95% interval confidence] (p value).

## Discussion

In this preclinical CMR study, we observed high test-retest reproducibility for left ventricular volumes and function with isoflurane anesthesia. Reproducibility was lower with pentobarbital anesthesia as well as for right ventricular parameters and ventricular strain analysis. The intra-study reproducibility assessed for tagged sequences was overall poor with both anesthetic procedures. Conversely, post-processing reproducibility, as evaluated by inter- and intra-observer comparisons, was good, with COV below 20% and positive and significant ICC values for most parameters assessed.

Our results highlight the influence of anesthesia on small-animal imaging of the heart. Differences in the cardiovascular effects of various anesthetic drugs are well known. Pentobarbital is a common short-acting barbiturate used for rodent anesthesia that induces different sleep times depending on mice strain, age, and sex^10^. It produces marked respiratory depression and heterogeneous cardiovascular effects depending on the animal species, dose used and expected duration of anesthesia. Indeed, two rat studies by Redfors B *et al*.^11^ and Stein A *et al*.^12^ showed that HR remained stable from baseline after anesthesia with pentobarbital 25–30 mg/kg IP, whereas HR was depressed with other anesthetic agents (isoflurane, ketamine/xylazine). In these two studies, left ventricular volumes were lower in animals anesthetized with pentobarbital (i.e., those with higher HR) than in animals anesthetized with isoflurane (i.e., those with depressed HR). By contrast, two other studies performed in mice showed that a higher dose of pentobarbital, 50–70 mg/kg IP, induced depressed HR from baseline, with a more pronounced effect with longer duration of anesthesia^13, 14^. Our findings are in agreement with these latter studies: a dose of pentobarbital 60 mg/kg IP, which is needed to maintain a sleep time of at least 60 min^10^, was associated with significantly lower HR compared with animals in the isoflurane group. In addition, mice in the pentobarbital group displayed significantly higher LVEDV than mice from the isoflurane group (62.3 ± 15.1 and 54.1 ± 6.2 µL, respectively; p < 0.05) (Table 2). Taken altogether, these data suggest that pentobarbital may have various cardiac effects, with higher pentobarbital doses and longer anesthesia leading to an HR drop, which favors greater diastolic ventricular filling and, consequently, left ventricular enlargement. A similar mechanism was suggested by Kober *et al*. in a study demonstrating that CMR measurements of left ventricular volumes were biased in mice anesthetized with ketamine/xylazine: this common anesthetic combination induced bradycardia and increased preload condition, resulting in higher left ventricular volumes compared with isoflurane anesthesia^15^. In addition to dose-related various pentobarbital cardiovascular effects, a versatile cardiovascular impact of this drug was also observed during long anesthesia, with a two-slope blood pressure pattern (initial decrease after anesthesia induction and secondary increase during anesthesia maintenance). In addition, the vasomotor effects of pentobarbital were less predictable (vasodilatation for some animals and vasoconstriction for others), with higher inter-individual variability of this parameter, compared with isoflurane^13^. These inconsistent results might be at least partially attributable to the low reproducibility of anesthesia depth, as suggested by the inter-study variation of BR observed in the present study, despite the use of identical doses of this drug. In addition, the cardiac effects of pentobarbital may vary depending on whether the gas inhaled during anesthesia is ambient air or a mixture, such as O2 + N2 or O2 + N2O^16^.

Isoflurane, the leading drug for inhaled anesthesia, is increasingly used in research settings (especially in the cardiovascular field) due to its reputation of neutrality with respect to cardiovascular parameters^17^. However, isoflurane may cause more pronounced respiratory depression than pentobarbital, as observed in the present study and by others^11^. In addition, some authors have reported vasodilatation properties of this halogenated ether, with potential impacts on blood flow^18, 19^. Iltis *et al*. also observed this effect and demonstrated that isoflurane anesthesia was associated with increased myocardial blood flow in Wistar rats compared with pentobarbital^16^. However, accumulating evidence favors the use of isoflurane in rodent cardiac imaging studies, such as a preclinical report highlighting the superiority of isoflurane anesthesia based on lower inter-subject variability of CMR left ventricular parameters compared with intraperitoneal anesthesia with MMF combination (medetomidine, midazolam and fentanyl)^20^. Our results provide additional evidence for the use of isoflurane to reduce the test-retest variability of left ventricular volume assessment when two CMR examinations are performed one week apart in the same healthy animals.

Conversely, in our trial, test-retest reproducibility of strain analysis, as measured by tissue tagging imaging, was not influenced by the anesthetic procedure and was low overall. However, tissue tagging remains the reference method for strain analysis in humans due to its high reproducibility, with test-retest COV below 10% for circumferential strain and below 20% for radial strain in healthy adults^5^. Similar results were obtained in patients with various pathological conditions in whom radial strain was less reproducible than circumferential strain^21^. Test-retest strain reproducibility with tissue tagging appears to be higher in humans compared with our mouse results. This difference might be explained by the higher temporal resolution of tagged sequences in human, which is inversely correlated with heart rate. Indeed, up to thirty tagged images were performed within the R-R interval for patients whose heart rate was 60–80 bpm, whereas in our study, given an approximate heart rate of 400 bpm in mice, a maximum of 14 images were acquired^5, 21^. In addition to this insufficient temporal sampling, the number of images per cardiac cycle was variable between two examinations or even within an exam session, depending on the mouse heart rate and the maximum number of images possible to acquire between two R-R. This might at least partly explain the test-retest and intra-study variability that we observed in our study for myocardial strain.

The implication of CMR acquisition parameters rather than post-processing issues is further supported by the high inter- and intra-observer reproducibility we observed for left ventricular parameters: analysis and re-analysis of the same data set by the same or another observer showed 5–10% COV. This result is consistent with Schneider J *et al*., who reported a 2–11% COV for inter- and intra-observer reproducibility after 11.2-T CMR mouse exams^22^. In our study, inter/intra-observer reproducibility was lower for right ventricular and radial strain assessment, with an increase in the COV to 20–30%. The lower reproducibility of radial strain has been discussed previously^5, 21^. Regarding right ventricular issues, there is a paucity of preclinical data, but a previous study showed lower reproducibility for RV evaluation compared with LV, given the complex geometry of the right heart and the challenging delineation of its cavities^23^. However, even for strain and RV parameters, ICC values were positive and significant for inter- and intra-observer evaluation, suggesting the robustness of CMR post-treatment in our process.

We acknowledge that our work has several limitations. First, we used only the sequences and software available at our research facility, without comparison with other methods. Ventricular function was assessed using a self-gating sequence (IntraGate®) based on a modified retrospectively gated sequence, which can introduce several biases with respect to how this process works, its validation and how the operator uses it. In addition, for post-processing, we used a post-processing software for ventricular cavities (Segment) and another for myocardial strain (OsiriX-intag). This latter software relies on local sine-wave modeling (SinMod) technology. A comparison between the results obtained with this software and with harmonic phase analysis (HARP) software might provide interesting insights regarding the poor strain reproducibility of our study. Such a comparison was performed by Miller *et al*. in patients with cardiomyopathy and in healthy subjects. They demonstrated that inter- and intra-observer variability were significantly higher with HARP compared with SinMod software, highlighting a potential role of the post-processing tool in reproducibility evaluation^24^. Another limitation of this work is the absence of standardized temporal sampling for strain sequences, secondary to physiological constraints, especially heart rate variability. Systematic use of the same temporal resolution might have decreased the variability of the results.

However, our study has several strengths. This study is the first CMR preclinical study to systematically explore inter-/intra-study and inter-/intra-observer reproducibility, thus permitting the differentiation of limits related to the acquisition or post processing of CMR images. In addition, no previous study has explored the differential effects of two anesthetic drugs on test-retest reproducibility, an important question for CMR, which is often used in a longitudinal approach. Finally, our research provides data sets for CMR in healthy mice.

In conclusion, preclinical CMR presented high reproducibility for the assessment of left ventricular function under isoflurane anesthesia. Right ventricular function and myocardial strain assessment were less reproducible, mainly due to physiological variability, whereas post-processing appeared robust.

## Methods

### Animals

All animal procedures conformed with the guidelines from Directive 2010/63/EU of the European Parliament on the protection of animals used for scientific purposes, and specific French laws were followed. All investigations and procedures were approved by the regional animals ethics committee (Cenomexa 054 - n°03854.01). Experiments were performed in 18–22-week-old male C57BL/6 mice weighing 25–30 g at baseline (Janvier Labs, Le Genest St Isle, France), which were housed individually in a temperature-controlled room with ad libitum access to standard mouse chow and water. Animals were randomized in two groups according to the anesthetic procedure applied to perform cardiac magnetic resonance: group IF (isoflurane anesthesia) and group P (pentobarbital anesthesia).

### Anesthetic procedures during CMR

Group IF: Anesthesia was induced and maintained with isoflurane (Baxter SAS, Maurepas, France) at concentrations of 5% and 1.5–3%, respectively. Isoflurane was delivered in an oxygen/nitrous oxide mix (0.6 L/min) via spontaneous breathing using a precision vaporizer. Isoflurane rate was titrated throughout the exam to maintain a 40–60/min breath rate (BR).

Group P: Anesthesia was induced and maintained with a 60 mg/kg intra-peritoneal (IP) injection of pentobarbital (CEVA, Libourne, France).

Heart rate (HR) (beats per min − bpm) and BR (inspirations per min − insp/min) were monitored every 15 minutes in all animals throughout all CMR examinations.

### Cardiac magnetic resonance (CMR)

**Figure 3:**
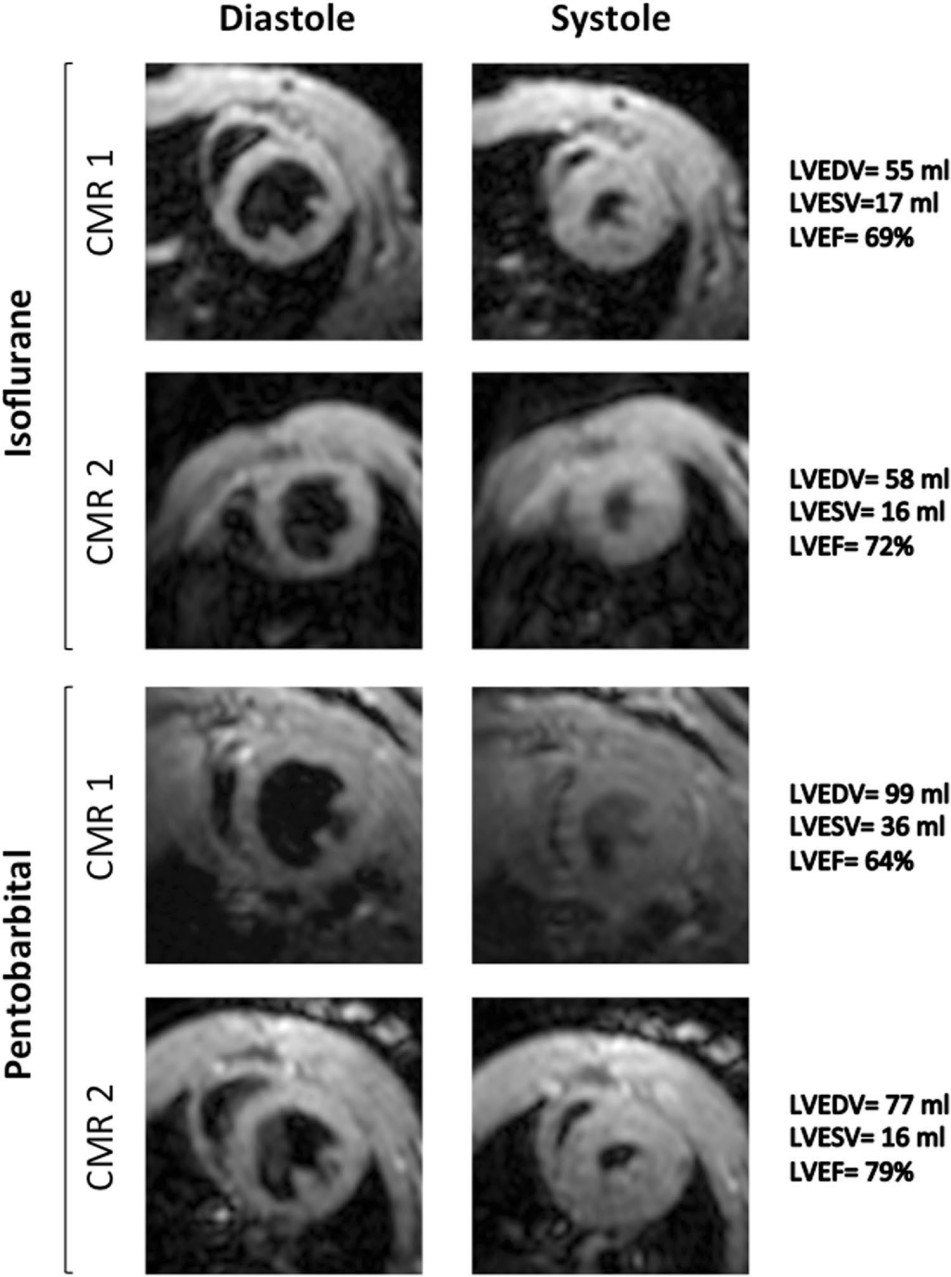
Sample systole and diastole images (black blood self-gated sequence IntraGate®) of repeated cardiac magnetic resonance (CMR1 and CMR2) in a mouse from group IF (upper four images) and in a mouse from group P (lower four images). Left ventricular parameters calculated from these exams (Segment® software) are indicated beside the images.

**Figure 4:**
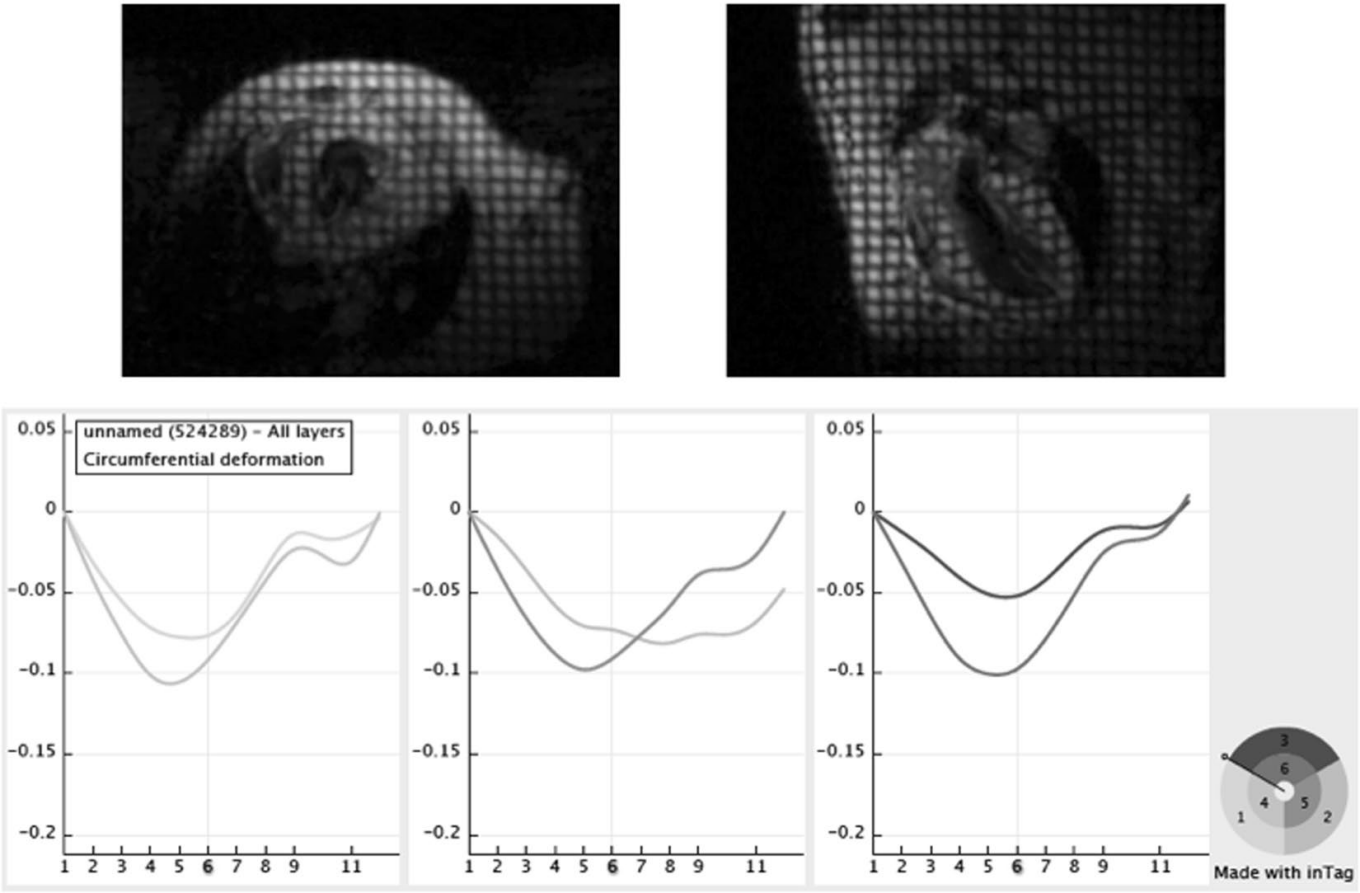
Sample short axis (upper left) and long axis (upper right) tagged CMR images, which were used to assess left ventricular strain. Sample circumferential strain time curves during cardiac cycle (lower panel) were obtained from short axis tagged images after post-processing using InTag plugin OsiriX® software.

In both groups, CMR was performed twice, a week apart (CMR1 and CMR2). A 7-T Bruker Pharmascan magnetic resonance (Bruker Biospin, Ettlingen, Germany) interfaced with a dedicated small-animal ECG and respiratory triggering system (SA Instruments) was used. A quadrature 1 H resonator was used for radiofrequency transmission (inner diameter = 72 mm) in conjunction with a surface single loop receive-only coil. Mice were placed in the supine position, and body temperature was maintained in a physiological range using a heating pad. Ventricular function was assessed using a black blood self-gated sequence (IntraGate®, Bruker Biospin, Ettlingen, Germany) based on a modified retrospectively gated Fast Low Angle Shot (FLASH) sequence [REF Hiba B 2006]. After multislab survey acquisition in 3 orthogonal axes (axial, coronal, and sagittal), 8 to 9 short axis IntraGate® slices ensuring a full coverage of the ventricles from the base to the apex were acquired (slice thickness 0.563 mm; echo time (TE): 2 ms; repetition time (TR): 5.5 ms; flip angle 25°; field of view: 28 × 28 mm^2^; matrix size: 128 × 128 mm^2^; spatial resolution: 0.219 × 0.219 mm^2^/pixel), with a temporal resolution of 16 images per cardiac cycle. Sample images of the self-gated sequence IntraGate® are shown on Fig. 3. Left ventricular strain was assessed using two short axis (basal third and apical third) and one long axis tagged CMR using a cine 2D Flash gated sequence (slice thickness 1 mm; TE/TR 3/8 ms; flip angle 15°). The assessment was performed twice within each CMR examination with 2 different matrices but the same spatial resolution to assess an intra-study reproducibility (first tagged acquisition: field of view 45 × 45 mm^2^; matrix size 256 × 256 mm^2^; spatial resolution 0.176 × 0.176 mm^2^/pixel; second tagged acquisition: field of view 22.4 × 22.4 mm^2^; matrix size 128 × 128 mm^2^; spatial resolution 0.175 × 0.175 mm^2^/pixel). Temporal resolution was variable depending on the maximum number of cardiac frames acquired during a single R-R interval (range, 10–14). Sample images of the tagged cine 2D Flash gated sequence are shown in Fig. 4.

### Cardiac function analysis

Ventricular parameters were analyzed in a blinded manner using Segment software after manual delineation of the right and left ventricular endocardial (excluding papillary muscles) and epicardial borders on all slices at end-diastole and end-systole (Segment v1.8 R1675, Medviso AB, University of Lund, Sweden). Left ventricular mass (LVM − µg), left/right ventricular ejection fraction (LVEF/RVEF − %) and left/right ventricular end diastolic and end systolic volume (LVEDV/RVEDV and LVESV/RVESV − µl) were therefore determined. Strain analysis was performed using the open source software OsiriX (http://www.osirix-viewer.com/) with the InTag plugin. Circumferential (Ecc) and radial (Err) strain was assessed on short axis (SA) frames, and longitudinal (Ell) strain was assessed on the long axis (LA) frame. Samples of strain time curves during the cardiac cycle (InTag plugin − OsiriX) are shown in Fig. 4.

### Reproducibility assessment

Test-retest reproducibility was assessed by comparing the results from the two CMR examinations (CMR1 and CMR2) performed one week apart. Intra-study reproducibility was assessed by comparing the results from the two tagged sequences performed within each CMR exam. For inter-observer reproducibility, each CMR exam was processed in a blinded manner by two different observers. For intra-observer reproducibility, a random subset of 5 CMR examinations was processed twice by the same observer, one week apart. For inter- and intra-study reproducibility, data were separately analyzed for each anesthetic procedure, as this parameter may influence the results.

### Statistical analysis

Quantitative data are expressed as the mean (standard deviation − SD). Reproducibility was evaluated by the mean (SD) absolute difference and coefficient of variation (COV) (mean relative difference) between results from CMR1 and CMR2, tagged sequence 1 and tagged sequence 2, observer 1 and observer 2 post-processing, and post-processing 1 and 2 of same examinations by the same observer for test-retest, intra-study, inter-observer and intra-observer reproducibility, respectively. A measurement was considered reproducible when COV was below 20%. Furthermore, the reproducibility was also assessed using the interclass correlation coefficient (ICC) and its 95% confidence interval (95%CI) under an ANOVA random effect model. As described by Tammemagi *et al*.^25^ the ICC can vary from −1 (perfect disagreement) to 0 (random agreement) and to +1 (perfect agreement). A negative ICC occurs when the between-subject variation is relatively small compared with the within-subject variation. An unpaired Student’s T-test was used for absolute differences comparisons between anesthetic procedures. HR, BR and body weight were compared within and between anesthetic procedures using paired and unpaired Student’s T-tests, respectively. A value of p < 0.05 was considered to denote significance. Ventricular parameters from CMR1 and CMR2 with both anesthetic procedures were also represented with Bland-Altman graphs, plotting the COV vs. average and the 95% limits of agreement. All tests were two tailed, and their level of significance (p) was defined as p < 0.05. IBM^®^-SPSS^®^ 22.0 for Windows^®^ was used as the statistical software.

## References

[1] Vallee JP, Ivancevic MK, Nguyen D, Morel DR, Jaconi M (2004). Current status of cardiac MRI in small animals. Magma..

[2] Caudron J (2013). MR relaxometry and perfusion of the myocardium in spontaneously hypertensive rat: correlation with histopathology and effect of anti-hypertensive therapy. Eur Radiol..

[3] Grothues F (2002). Comparison of interstudy reproducibility of cardiovascular magnetic resonance with two-dimensional echocardiography in normal subjects and in patients with heart failure or left ventricular hypertrophy. Am J Cardiol..

[4] Grothues F (2004). Interstudy reproducibility of right ventricular volumes, function, and mass with cardiovascular magnetic resonance. Am Heart J..

[5] Swoboda PP (2014). Reproducibility of myocardial strain and left ventricular twist measured using complementary spatial modulation of magnetization. J Magn Reson Imaging..

[6] Hiba B, Richard N, Janier M, Croisille P (2006). Cardiac and respiratory double self-gated cine MRI in the mouse at 7 T. Magn Reson Med..

[7] Vanhoutte L (2015). Variability of mouse left ventricular function assessment by 11.7 Tesla MRI. J Cardiovasc Trans Res..

[8] Wu EX, Towe CW, Tang H (2002). MRI cardiac tagging using a sinc-modulated RF pulse train. Magn Reson Med..

[9] Zhou R, Pickup S, Glickson JD, Scott CH, Ferrari VA (2003). Assessment of global and regional myocardial function in the mouse using cine and tagged MRI. Magn Reson Med..

[10] Lovell DP (1986). Variation in barbiturate sleeping time in mice. 3. Strain x environment interactions. Lab Anim..

[11] Redfors B, Shao Y, Omerovic E (2014). Influence of anesthetic agent, depth of anesthesia and body temperature on cardiovascular functional parameters in the rat. Lab Anim..

[12] Stein AB (2007). Effects of anesthesia on echocardiographic assessment of left ventricular structure and function in rats. Basic Res Cardiol..

[13] Matsuda Y (2007). Comparison of newly developed inhalation anesthesia system and intraperitoneal anesthesia on the hemodynamic state in mice. Biol Pharm Bull..

[14] Janssen BJ (2004). Effects of anesthetics on systemic hemodynamics in mice. Am J Physiol Heart Circ Physiol..

[15] Kober F, Iltis I, Cozzone PJ, Bernard M (2004). Cine-MRI assessment of cardiac function in mice anesthetized with ketamine/xylazine and isoflurane. MAGMA..

[16] Iltis I (2005). *In vivo* assessment of myocardial blood flow in rat heart using magnetic resonance imaging: effect of anesthesia. J Magn Reson Imaging..

[17] Hildebrandt IJ, Su H, Weber WA (2008). Anesthesia and other considerations for *in vivo* imaging of small animals. ILAR J..

[18] Haensel JX, Spain A, Martin C (2015). A systematic review of physiological methods in rodent pharmacological MRI studies. Psychopharmacology..

[19] Du C (2009). Differential effects of anesthetics on cocaine’s pharmacokinetic and pharmacodynamic effects in brain. Eur J Neurosci..

[20] Grabmaier U (2014). The role of 1.5 tesla MRI and anesthetic regimen concerning cardiac analysis in mice with cardiomyopathy. PLoS One..

[21] Donekal S (2013). Inter-study reproducibility of cardiovascular magnetic resonance tagging. J Cardiovasc Magn Reson..

[22] Schneider JE (2003). Fast, high-resolution *in vivo* cine magnetic resonance imaging in normal and failing mouse hearts on a vertical 11.7 T system. J Magn Reson Imaging..

[23] Wiesmann F (2002). Analysis of right ventricular function in healthy mice and a murine model of heart failure by *in vivo* MRI. Am J Physiol Heart Circ Physiol..

[24] Miller CA (2013). Comparison of local sine wave modeling with harmonic phase analysis for the assessment of myocardial strain. J Magn Reson Imaging..

[25] Tammemagi MC, Frank JW, Leblanc M, Artsob H, Streiner DL (1995). Methodological issues in assessing reproducibility–a comparative study of various indices of reproducibility applied to repeat ELISA serologic tests for Lyme disease. J Clin Epidemiol..

